# Efficacy and Safety of the Radiotherapy for Liver Cancer: Assessment of Local Controllability and Its Role in Multidisciplinary Therapy

**DOI:** 10.3390/cancers12102955

**Published:** 2020-10-13

**Authors:** Marina Ohkoshi-Yamada, Kenya Kamimura, Osamu Shibata, Shinichi Morita, Motoki Kaidu, Toshimichi Nakano, Katsuya Maruyama, Atsushi Ota, Hirotake Saito, Nobuko Yamana, Tomoya Oshikane, Yukiyo Goto, Natsumi Yoshimura, Satoshi Tanabe, Hisashi Nakano, Madoka Sakai, Yuto Tanaka, Yohei Koseki, Yoshihisa Arao, Hiroyuki Abe, Toru Setsu, Akira Sakamaki, Takeshi Yokoo, Hiroteru Kamimura, Hidefumi Aoyama, Shuji Terai

**Affiliations:** 1 Division of Gastroenterology and Hepatology, Graduate School of Medical and Dental Sciences, Niigata University, 1-757 Asahimachido-ri, Chuo-ku, Niigata, Niigata 951-8510, Japan; marinao@med.niigata-u.ac.jp (M.O.-Y.); oshibatai@med.niigata-u.ac.jp (O.S.); s-morita@med.niigata-u.ac.jp (S.M.); ytanaka@med.niigata-u.ac.jp (Y.T.); ykoseki@med.niigata-u.ac.jp (Y.K.); y-arao@med.niigata-u.ac.jp (Y.A.); hiroyukiabe@med.niigata-u.ac.jp (H.A.); setsut@med.niigata-u.ac.jp (T.S.); saka-a@med.niigata-u.ac.jp (A.S.); t-yokoo@med.niigata-u.ac.jp (T.Y.); hiroteruk@med.niigata-u.ac.jp (H.K.); terais@med.niigata-u.ac.jp (S.T.); 2 Department of Radiology and Radiation Oncology, Niigata University Graduate School of Medical and Dental Sciences, 1-757 Asahimachido-ri, Chuo-ku, Niigata, Niigata 951-8510, Japan; kaidu@med.niigata-u.ac.jp (M.K.); prague2657@yahoo.co.jp (T.N.); maruyama@niigata-nogeka.or.jp (K.M.); aohta@med.niigata-u.ac.jp (A.O.); 1rtkcyte@med.niigata-u.ac.jp (H.S.); n.yamana99@gmail.com (N.Y.); gripen.med@gmail.com (T.O.); yukiyogoto15@gmail.com (Y.G.); na.kamiya1027@gmail.com (N.Y.); s-tanabe@med.niigata-u.ac.jp (S.T.); nakanoh@med.niigata-u.ac.jp (H.N.); madoka.sakai.18@gmail.com (M.S.); h-aoyama2019@med.hokudai.ac.jp (H.A.); 3 Department of Radiation Oncology, Faculty of Medicine, Hokkaido University, Kita 15-jo Nishi 7-chome, Kita-ku, Sapporo, Hokkaido 060-8638, Japan

**Keywords:** hepatocellular carcinoma, radiotherapy, Child–Pugh score, ALBI score, local disease control, portal vein tumor thrombus

## Abstract

**Simple Summary:**

We have shown the efficacy and safety of radiotherapy for the hepatocellular carcinoma as a curative therapy and a part of multidisciplinary therapy which could safely be combined with various therapies. The assessment of local controllability and survival showed the anti-tumor efficacy of the procedure, and the analyses of the serum biochemical factors and hepatic function, including the Child–Pugh score and albumin–bilirubin (ALBI)-grade, demonstrated its safety. While the assessment of a larger number of cases and well-designed randomized trials are essential, the results provided here will contribute to understanding the efficacy of radiotherapy as an additional therapy for the treatment of hepatocellular carcinoma and help physicians make therapeutic decisions.

**Abstract:**

This study investigated the efficacy and safety of radiotherapy as part of multidisciplinary therapy for advanced hepatocellular carcinoma (HCC). Clinical data of 49 HCC patients treated with radiotherapy were assessed retrospectively. The efficacy of radiotherapy was assessed by progression-free survival, disease control rate, and overall survival. Safety was assessed by symptoms and hematological assay, and changes in hepatic reserve function were determined by Child–Pugh score and albumin–bilirubin (ALBI) score. Forty patients underwent curative radiotherapy, and nine patients with portal vein tumor thrombus (PVTT) underwent palliative radiotherapy as part of multidisciplinary therapy. Local disease control for curative therapy was 80.0% and stereotactic body radiotherapy was 86.7% which was greater than that of conventional radiotherapy (60.0%). Patients with PVTT had a median observation period of 651 days and 75% three-year survival when treated with multitherapy, including radiotherapy for palliative intent, transcatheter arterial chemoembolization, and administration of molecular targeted agents. No adverse events higher than grade 3 and no changes in the Child–Pugh score and ALBI score were seen. Radiotherapy is safe and effective for HCC treatment and can be a part of multidisciplinary therapy.

## 1. Introduction

Various conventional therapeutic options, including surgery, radiofrequency ablation (RFA), transarterial chemoembolization (TACE), chemotherapy including molecular targeting agents (MTA), and immunotherapy are used in combination to treat hepatocellular carcinoma (HCC). Therapeutic decisions are made and several algorithms have been established based on assessment of performance status, hepatic reserve function, and disease stage [1]. Recently, the number of elderly HCC patients who are treated with anticoagulant and antiplatelet therapy for complications of cerebral or cardiovascular diseases and have difficulty breathing is significantly increasing. The presence of these complications affects decision-making for therapeutic options in these patients [2], and there is an unmet need for effective and less invasive therapy. There have been significant advances in the use of radiotherapy for liver tumors, and the number of patients treated with stereotactic body radiation therapy (SBRT) is increasing [3,4,5,6]. However, due to limited evidence reported to date, SBRT has not been described as a standard therapy in the guidelines [1].

Based on its promising effects, the number of HCC patients treated with radiation therapy is increasing. To clarify its efficacy and safety, we have summarized the clinical courses of patients with HCC treated with radiotherapy in our hospital and assessed the disease controllability and safety aspects considering hepatic function from the standpoint of hepatologists. This summary will help physicians to decide the therapeutic options for HCC, especially when RFA is unavailable and patients with advanced stages for both local disease control and as part of multidisciplinary therapy.

## 2. Methods

### 2.1. Patients

This retrospective study was designed as a noninterventional, observational study. The study was approved for the data collection by the ethics committee and institutional review board of Niigata University Hospital (#2192, 2020-0142). Either written informed consent or the Opt-out method were used to obtain the agreement for the data collection from all patients, and the study was conducted in accordance with the ethical guidelines of the 1975 Declaration of Helsinki. Decisions for therapeutic options, including radiotherapy, were made based on the algorithms [1] by the multidisciplinary liver tumor boards of Niigata University, and the radiotherapy was performed using Novalis (Brainlab AG, Munich, Germany). The total dose to the planning target volume (PTV) was described by the biological effective dose (BED) and equivalent dose in 2Gy fractions (EQD2), using the α/β ratio which is 10 for HCC treatment.

### 2.2. Evaluation of Antitumor Effects

The prognosis of the patients was obtained based on the clinical records. Antitumor response was evaluated from either computed tomography (CT) or magnetic resonance imaging (MRI) images obtained before and 3 months after treatment. Evaluation was performed in accordance with the modified Response Evaluation Criteria in Solid Tumors (RECIST) guideline. The disease control rate (DCR) was defined as the percentage of patients achieving complete response, partial response, and stable disease. The tumor markers alpha-fetoprotein (AFP) and des-gamma-carboxy prothrombin (DCP) were followed at appropriate time periods for each patient.

### 2.3. Evaluation of Safety

Hematological and nonhematological toxicity in all patients was evaluated using NCI-CTCAE (National Cancer Institute Common Terminology Criteria for Adverse Events) version 4.0. To evaluate the impact of the treatment on general condition, hematological assessments were performed before, immediately after, and 3 months after radiotherapy, and the values were expressed as percentages of the initial values. Hepatic function was assessed by the Child–Pugh score and the albumin–bilirubin (ALBI) score before, right after (within a week), and 3 months after irradiation.

### 2.4. Statistical Analysis

Progression-free survival (PFS) was assessed by the Kaplan–Meier method. Time-dependent changes in serum biochemical assays, Child–Pugh score, and ALBI score were analyzed by the Kruskal–Wallis test followed by Dunn’s multiple comparison test using the GraphPad Software (GraphPad, LaJolla, CA, USA). *p*-values less than 0.05 were considered to indicate a significant difference.

## 3. Results

### 3.1. Patient Characteristics

**Figure 1:**
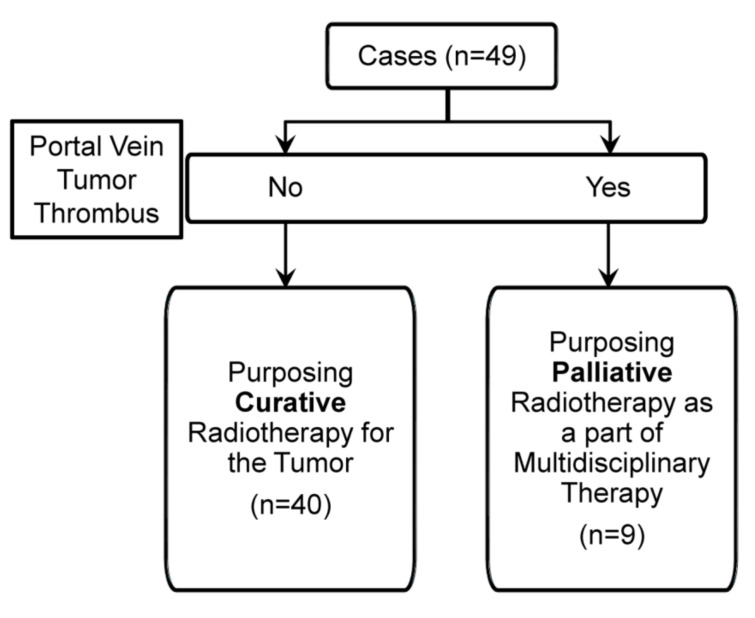
Distinction of the patients.

**Figure 2:**
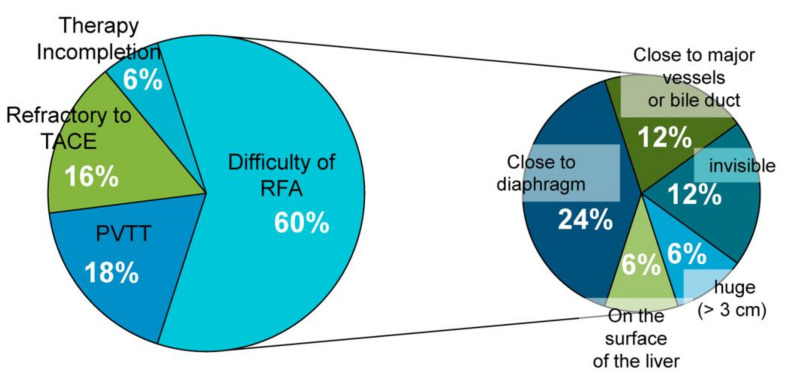
Reasons for choosing radiotherapy. TACE, transarterial chemoembolization, PVTT, portal vein tumor thrombosis, RFA, radiofrequency ablation.

During the period of 2013–2019, 54 patients were treated with radiotherapy. Among them, the clinical course, symptoms and laboratory data were available for 49 cases, and efficacy and safety of the radiotherapy were assessed. Among the 49 patients, 40 patients underwent curative radiotherapy (the curative group), and nine patients with complications of portal vein tumor thrombosis underwent palliative radiotherapy (the palliative group) as a part of multidisciplinary therapy (Figure 1). Table 1 shows the clinical characteristics of these patients. The median age was 71 years (range, 44 to 86) and 71 years (range, 50 to 84) for the curative and palliative groups, respectively. The curative group included seven patients with Child–Pugh grade B, and the median ALBI score was −2.39 (range, −3.21 to −1.50) before treatment. The palliative group included one patient with Child–Pugh grade B, and the median ALBI score was −2.25 (range, −2.90 to −1.50) before treatment. The remaining patients were of Child–Pugh grade A. In the curative group, six patients were in Barcelona clinic liver cancer (BCLC) disease stage 0, 26 patients were in stage A, and eight patients were in stage B; all nine patients in the palliative group were in stage C. No extrahepatic metastases were found in any patient. All patients with portal vein tumor thrombus (PVTT) in the peripheral branch underwent conventional fractionated radiotherapy with a radiation dose of 46.0 (32.5–50.0) Gy (equivalent dose in 2Gy fractions, EQD2). Eleven of 40 patients in the curative group underwent conventional radiotherapy with a dosage of 50.0 (32.5–77) Gy (equivalent dose in 2Gy fractions, EQD2); the remaining 29 patients, who were mainly recent patients, were treated with stereotactic body radiation therapy (SBRT) with a dosage of 65.0 (50.0–77.0) Gy (equivalent dose in 2Gy fractions, EQD2). The curative group, especially SBRT group, showed a higher biological effective dose (BED) and equivalent dose in 2Gy fractions (EQD2) values to the planning target volume (PTV) and a lower mean liver dose as well as the liver volume receiving 30Gy or greater (V30Gy), suggesting the efficacy and safety of SBRT (Table 1). In the curative group, 22 patients underwent prior treatment with TACE and RFA for the targeted lesion, and 23 patients were treated with TACE, RFA, and MTA following radiotherapy for recurrent lesions in the liver, including new lesions (Table 1). The reasons for choosing radiotherapy included incompletion of the therapies (6%), refractoriness to TACE (16%), complications of PVTT (18%), and difficulty with RFA (60%). The most common reasons for difficulty with RFA were the position of the tumor, tumor size, and invisibility of the tumor (Figure 2).

### 3.2. Efficacy

#### 3.2.1. Curative Radiation

**Figure 3:**
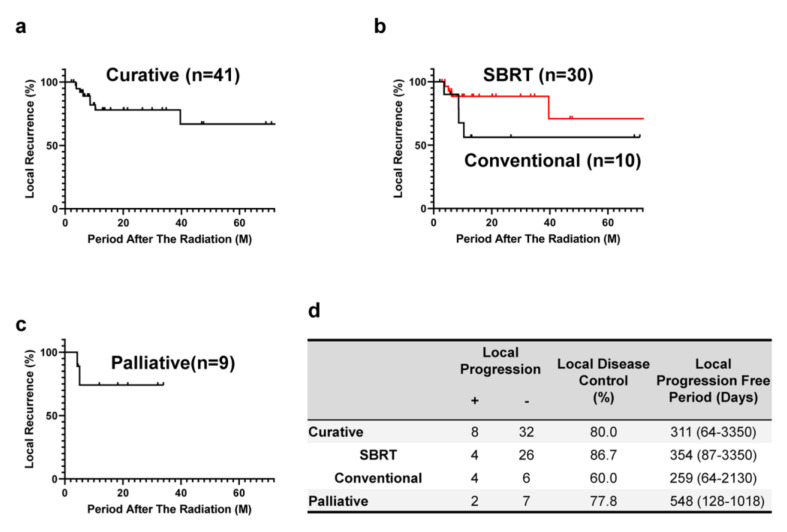
Effect of radiotherapy on local disease control. (**a**) Local progression-free survival (PFS) of patients treated with radiotherapy aimed at curative therapy (*n* = 40). (**b**) Local PFS of patients treated with conventional radiotherapy (*n* = 10, black solid line) and SBRT (*n* = 30, red solid line). (**c**) Local PFS of patients treated with radiotherapy aimed at palliative therapy (*n* = 9). M, months; SBRT, stereotactic body radiation therapy. (**d**) Summary of local disease control.

**Figure 4:**
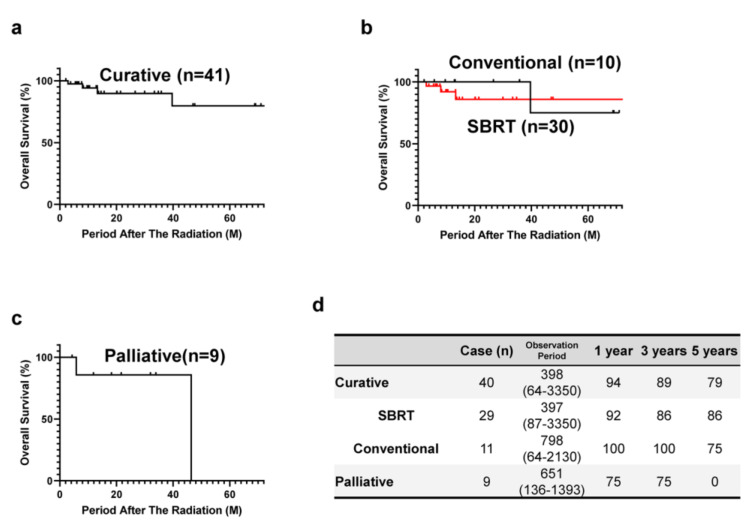
Effect of radiotherapy on survival. (**a**) Overall survival (OS) of patients treated with radiotherapy aimed at curative therapy (*n* = 40). (**b**) OS of patients treated with conventional radiotherapy (*n* = 10, black solid line) and SBRT (*n* = 30, red solid line). (**c**) OS of patients treated with radiotherapy aimed at palliative therapy (*n* = 9). M, months; SBRT, stereotactic body radiation therapy. (**d**) Summary of survival periods.

In all cases in which curative radiation was performed, the median PFS was 311 (64–3350) days; the median PFS was 354 (87–3350) days in the SBRT group and 259 (64–2130) days in the conventional fractionated radiotherapy group. The local control rate was 80.0% for overall curative radiation, 86.7% for the SBRT group and 60.0% for the conventional radiotherapy group (Figure 3). The five-year survival rate was 79% for overall curative radiation, 86% for the SBRT group and 75% for the conventional radiation group although no statistical differences were marked (Figure 4).

#### 3.2.2. Palliative Radiation

**Figure 5:**
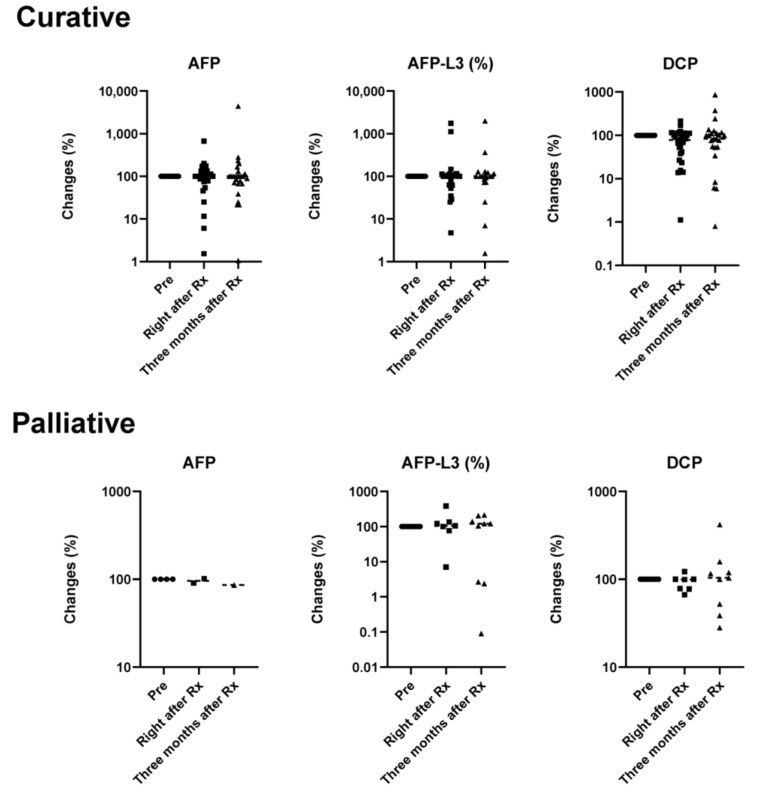
Effect of radiotherapy on tumor markers. Changes (%) of tumor markers compared with values before treatment. Black solid circles, squares, and triangles represent data for before (Pre), right after Rx, and three months after Rx, respectively. AFP, alpha-fetoprotein; AFP-L3, L3 isoform of AFP; DCP, des-gamma-carboxy prothrombin; Rx, radiotherapy.

The nine patients with complications of PVTT had a median PFS of 548 (128–1018) days and a local DCR of 77.8%. The three-year survival rate was 75% with various combinations of treatments (Table 1 and Table 2). Importantly, local control of the tumor was achieved in six patients and various therapeutic options were combined after palliative radiotherapy. These six patients received TACE, and five of them were further treated with MTA (Table 2). These patients had no history of hospitalization due to hepatic failure or other adverse events, indicating the contribution of radiotherapy to their quality of life. The tumor markers alpha-fetoprotein (AFP), L3 isoform of AFP (AFP-L3), and DCP were followed before, immediately after, and three months after radiotherapy (Figure 5). The values showed no significant changes due to variability in each case; however, the overall tendency showed a decrease in the tumor markers after radiotherapy in both groups (Figure 5).

### 3.3. Safety

The haematological and nonhaematological toxicities were evaluated for each treatment cycle using NCI-CTCAE version 4.0. No grade ≥2 hematological or nonhematological adverse events were seen. There were no treatment-related deaths.

**Figure 6:**
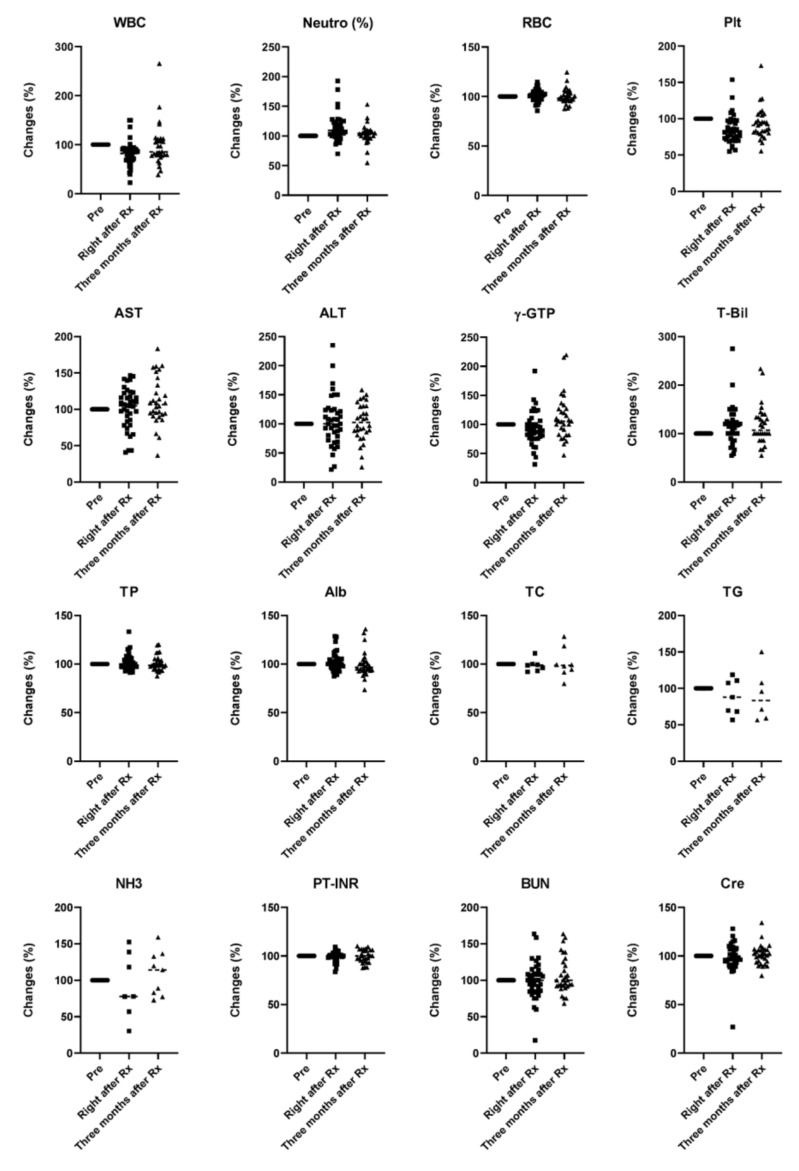
Safety of curative radiotherapy for hepatocellular carcinoma. Data represent the changes from initial values. Black solid circles, squares, and triangles represent data for before (Pre), right after Rx, and three months after Rx, respectively. WBC, white blood cell count; neutrophils (%); RBC, red blood cell count; plt, platelet count, AST, aspartate aminotransferase; ALT, alanine aminotransferase; γ-GTP, γ-glutamyltransferase; T-Bil, total bilirubin; TP, total protein; Alb, albumin; TC, total cholesterol; TG, triglyceride; NH3, ammonia; PT-INR, international normalized ratio of prothrombin time; BUN, blood urea nitrogen; Cre, creatinine. Kruskal–Wallis test followed by Dunn’s multiple comparison test.

**Figure 7:**
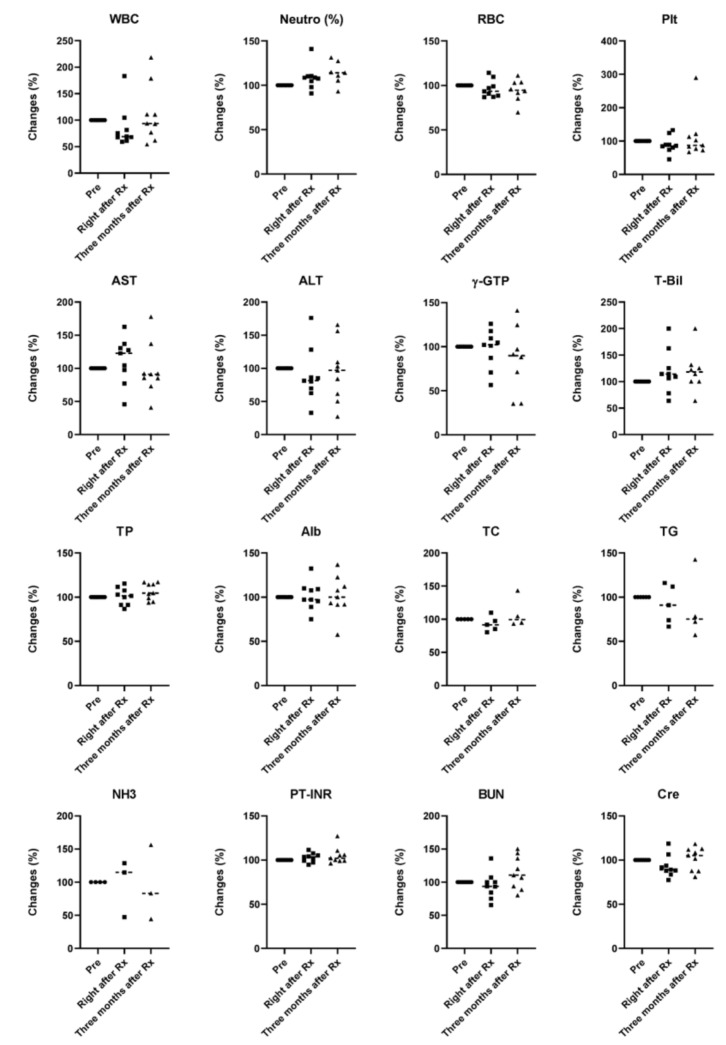
Safety of palliative radiotherapy for hepatocellular carcinoma. Data represent the changes from initial values. Black solid circles, squares, and triangles represent data for before (Pre), right after Rx, and three months after Rx, respectively.

**Figure 8:**
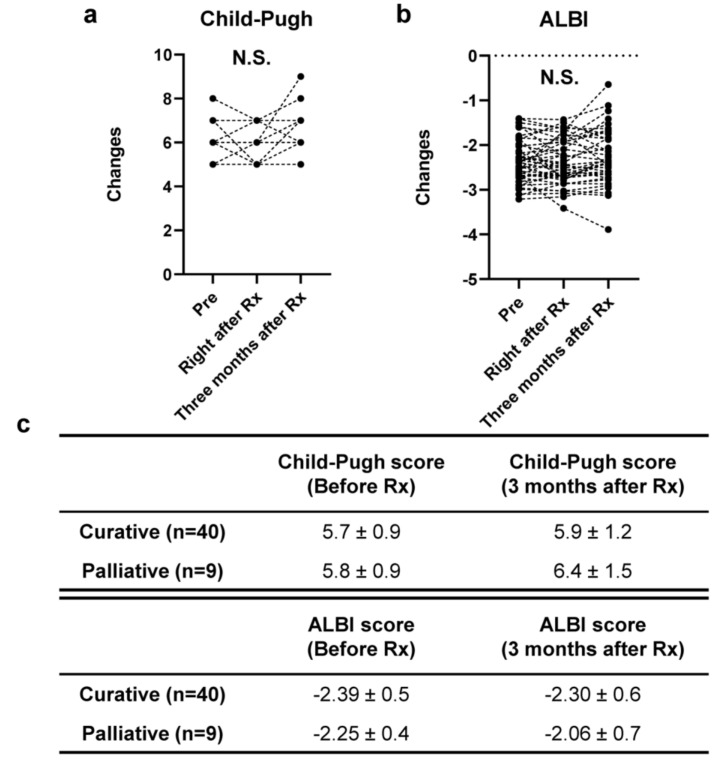
Effect of radiotherapy for hepatocellular carcinoma on hepatic function. Data represent changes in Child–Pugh score (**a**) and albumin–bilirubin (ALBI) score (**b**) for each patient. The median values were statistically analyzed with the Kruskal–Wallis test followed by Dunn’s multiple comparison test. (**c**) Summary of the values before and 3 months after radiotherapy.

A careful follow-up of peripheral blood and biochemical findings for all patients, including white blood cell count (WBC), neutrophils (%), red blood cell count (RBC), platelet count (plt), aspartate aminotransferase (AST), alanine aminotransferase (ALT), γ-glutamyltransferase (γ-GTP), total bilirubin (T-Bil), total protein (TP), albumin (Alb), total cholesterol (TC), triglycerides (TG), ammonia (NH_3_), international normalized ratio of prothrombin time (PT-INR), blood urea nitrogen (BUN), and creatinine (Cre), was performed to monitor the patients’ general condition and the toxicity of curative (Figure 6) and palliative (Figure 7) radiotherapy. Although a tendency for an increase in the Child–Pugh score after radiation was seen in the palliative group, there were no significant changes in the Child–Pugh score or ALBI score (Figure 8).

## 4. Discussion

Our results showed the safety and efficacy of both curative and palliative radiotherapy for HCC. The local DCR for curative radiotherapy in our hospital was 80% in total and 86.7% for SBRT. The results were consistent with the results of a few clinical studies focusing on the effectiveness of radiotherapy for local treatment of HCC [7,8,9,10,11]. Hara et al. reported that the three-year local recurrence rate was significantly lower with radiotherapy than with RFA (5.3% vs. 12.9%) in patients with well-compensated liver function [9]. Wong et al. reported the efficacy of SBRT utilizing the propensity score matching procedure to compare the prognosis of the TACE + SBRT group with that of the TACE alone group. Median survival and median PFS were better in the TACE + SBRT group than in the TACE alone group (23.9 vs. 10.4 months and 7.6 months vs. 5.7 months), respectively [10]. In addition, the radiological DCR was better in the TACE + SBRT group than in the TACE alone group (98% vs. 56.7%) [10]. Furthermore, the efficacy of TACE + SBRT was comparable to the previously reported local control rate of 88% following the combination of TACE + RFA [12]. In addition, Fu et al. reported the efficacy of SBRT as a salvage therapy after the incomplete RFA [13]. Recently, Bettinger et al. reported that the therapeutic effect of radiotherapy was comparable to that of sorafenib [14]. These results support the efficacy of radiotherapy for HCC as a curative therapy.

We have also demonstrated the safety and efficacy of radiotherapy for PVTT-complicated HCC cases. PVTT is a poor prognostic factor, and the choice of therapeutic strategy among surgical resection, TACE, radiotherapy, and chemotherapy has not been established [15,16,17]. Our results showed that HCC patients with PVTT treated with radiotherapy achieved a three-year survival rate of 75%. This is greater than the previously reported one-year overall survival rates of 30.9% and 9.2% and two-year overall survival rates of 3.8% and 0% for the TACE group and the conservatively treated group, respectively (*p* < 0.001), showing the efficacy of TACE [18]. Recently, MTA have been applied for HCC treatment; however, studies in the Asia-Pacific region reported that sorafenib had an objective response rate of less than 5% and a median time to progression of 2.8 months, showing insufficient efficacy [19]. A few clinical studies have reported on the usefulness of RT for PVTT cases. Li et al. compared a TACE + RT group with a TACE alone group in 216 patients with HCC complicated by PVTT and found significantly prolonged overall survival in the TACE + RT group (10.9 vs. 4.1 months, *p* < 0.001), suggesting the antitumor effect of radiotherapy for PVTT [20]. In addition, Tang et al. reported that among 371 patients with resectable HCC with PVTT, those who were treated by radiotherapy had a median overall survival of 2.3 months longer than those who were treated with surgical resection (*p* = 0.03) [21]. Recently, Wei et al. have shown the efficacy of radiotherapy as a neoadjuvant therapy for resectable HCC which provided the better postoperative survival outcomes than surgery alone [22]. These reports support the results in this study, however, due to the small number of cases assessed in the current study. Further studies with a larger number of cases are essential to compare the antitumor effect to that of TACE, RFA, and MTA.

Adverse events of anorexia, nausea, exacerbation of liver damage, and bone marrow suppression are often reported after radiotherapy, and most of these events are resolved within a few weeks [23,24]. Li et al. reported that no patients treated with radiotherapy experienced any serious adverse events necessitating discontinuation of the radiation [20]. Radiation-induced liver disease (RILD) has gained attention recently because of concerns for the safety of radiotherapy. Compared with classic RILD due to dose-limiting complications, non-classic RILD, which occurs with radiotherapy using CT-based planning, causes increases in transaminase and bilirubin [23,24]. Our patients showed no significant adverse events after radiotherapy [20]. The safety of the treatment was also assessed by the Child–Pugh score and the ALBI score before, right after (within a week), and three months after irradiation, which showed no changes after the therapy. This effect resulted in the successful combination of multidisciplinary therapy, as reported [25]. To date, the usefulness of the Child–Pugh score after the radiotherapy has been reported, and its increase of points is important to estimate the occurrence of RILD [26]. Indeed, it is also evidenced in our study that any of our cases showed increase of Child–Pugh score higher than 2 points and no significant RILD was seen. This is probably due to the planning of the radiotherapy focusing on the safety as our study involved a total of eight cases of Child B class. It is evidenced by the lower mean liver dose and volume receiving 30Gy or greater (V30Gy) of the liver with higher biological effective dose (BED) and equivalent dose in 2Gy fractions (EQD2) to the planning target volume (PTV) in the curative group (Table 1). The ALBI score is a simple and objective liver function score based on albumin and bilirubin values and has been reported to be useful in assessing liver function in HCC treatment including SBRT [27,28,29]. The results of ALBI scores obtained in this study in a time-dependent manner after radiotherapy for HCC, although the number of cases was small, are a novel finding showing the safety of the procedure. The mild worsening of the scores in the palliative group might have been due to tumor growth. However, compared to the requirement of the Child–Pugh grade of A for MTAs and unexpected effect on the hepatic reserve function of the MTAs and TACE, the radiotherapy can be a safe and effective therapeutic option for either curative and palliative therapy for the cases with poorer hepatic reserve function [1,30,31]. It is also clear that multidisciplinary boards, including hepatologists, oncologists, radiologists, and surgeons, should discuss the therapeutic decisions [32] as the various therapeutic options are available for HCC, currently [33].

The limitations of the present study are the small number of cases, the retrospective observational design of the study, and difficulty showing the statistical significance. Based on the promising results, assessment of a larger number of cases, well-designed randomized trials, and comparison with the conventional therapies are essential to further propose the importance of the radiotherapy.

## 5. Conclusions

Radiation therapy for HCC is safe and effective as a locoregional treatment and can be safely and successfully combined with other treatments.

## Figures and Tables

**Table 1:** Characteristics of patients.

Characteristic	Curative Group	Palliative Group
Number of patients	40	9
Age (yr)	71 (44–86)	71 (50–84)
Gender (M:F)	30:10	6:3
Etiology (HBV, HCV, NBNC)	3, 18, 19	4, 2, 3
Child–Pugh (A, B, C)	33, 7, 0	8, 1, 0
ALBI score	−2.39 (−3.21 to −1.50)	−2.25 (−2.90 to −1.50)
BCLC staging (0, A, B, C)	6, 26, 8, 0	0, 0, 0, 9
Recurrence (Yes/No)	16/24	3/6
Types of radiotherapy		
SBRT	29	0
Conventional	11	9
Total dose (Gy)	SBRT 51 (44–60) Conventional 50 (30–50)	48 (30–50)
Dose per fraction (Gy)	SBRT 3 (2–11) Conventional 2 (2–3 Gy)	2 (2–3)
BED to the PTV	SBRT 78.0 (60.0–92.4) Conventional 60.0 (39.0–66.3)	55.2 (39.0–60.0)
EQD2 to the PTV	SBRT 65.0 (50.0–77.0) Conventional 50.0 (32.5–77.0)	46.0 (32.5–50.0)
Mean liver dose (Gy)	SBRT 11.7 (3.3–19.3) Conventional 14.7 (4.1–21.9)	12.6 (8.2–18.7)
V30Gy of the liver	SBRT 11.4 (3.5–28.4) Conventional 18.0 (1.4–40.4)	19.7 (12.3–34.0)
Pre-treatment		
TACE	17	4
RFA	5	0
MTA	0	2
Post-treatment		
TACE	16	6
RFA	1	0
MTA	6	5

M, male; F, female; HBV, hepatitis B virus infection; HCV, hepatitis C virus infection; NBNC, non-B, non-C virus induced liver disease; BCLC, Barcelona clinic liver cancer staging; SBRT, stereotactic body radiation therapy; BED, biological effective dose; PTV, planning target volume; EQD2, equivalent dose in 2Gy fractions; V30Gy, the liver volume receiving 30Gy or greater, TACE, transarterial chemoembolization; RFA, radiofrequency ablation; MTA, molecular targeted agents.

**Table 2:** Summary of the post radiotherapy for cases with PVTT.

Case Number	TACE (Times)	MTA
1	1	SOR
2	3	SOR
3	1	LEN
4	6	LEN
5	1	SOR
6	2	-

TACE, transarterial chemoembolization; MTA, molecular targeted agents; SOR, sorafenib; LEN, lenvatinib.
